# Biliary Neuroendocrine Neoplasms: Analysis of Prognostic Factors and Development and Validation of a Nomogram

**DOI:** 10.3389/fonc.2021.654439

**Published:** 2021-07-19

**Authors:** Shengnan Zhou, Shitao Jiang, Weijie Chen, Haixin Yin, Liangbo Dong, Hao Zhao, Shaoqi Han, Xiaodong He

**Affiliations:** ^1^ General Surgery Department, Peking Union Medical College Hospital, China Academy of Medical Science & Peking Union Medical College, Beijing, China; ^2^ Liver Surgery Department, Peking Union Medical College Hospital, China Academy of Medical Science & Peking Union Medical College, Beijing, China

**Keywords:** biliary neuroendocrine neoplasms, nomogram, prognostic factors, survival rate, SEER database

## Abstract

**Background:**

For this study, we explored the prognostic profiles of biliary neuroendocrine neoplasms (NENs) patients and identified factors related to prognosis. Further, we developed and validated an effective nomogram to predict the overall survival (OS) of individual patients with biliary NENs.

**Methods:**

We included a total of 446 biliary NENs patients from the SEER database. We used Kaplan-Meier curves to determine survival time. We employed univariate and multivariate Cox analyses to estimate hazard ratios to identify prognostic factors. We constructed a predictive nomogram based on the results of the multivariate analyses. In addition, we included 28 biliary NENs cases from our center as an external validation cohort.

**Results:**

The median survival time of biliary NENs from the SEER database was 31 months, and the value of gallbladder NENs (23 months) was significantly shorter than that of the bile duct (45 months) and ampulla of Vater (33.5 months, p=0.023). Multivariate Cox analyses indicated that age, tumor size, pathological classification, SEER stage, and surgery were independent variables associated with survival. The constructed prognostic nomogram demonstrated good calibration and discrimination C-index values of 0.783 and 0.795 in the training and validation dataset, respectively.

**Conclusion:**

Age, tumor size, pathological classification, SEER stage, and surgery were predictors for the survival of biliary NENs. We developed a nomogram that could determine the 3-year and 5-year OS rates. Through validation of our central database, the novel nomogram is a useful tool for clinicians in estimating individual survival among biliary NENs patients.

## Introduction

Neuroendocrine neoplasms represent a group of highly heterogeneous diseases (depending on the primary site) and originate from peptidergic neurons and neuroendocrine cells (1). Gastroenteropancreatic neuroendocrine neoplasms (GEP-NENs) account for approximately 55% of all NENs. However, according to the European Neuroendocrine Tumour Society (ENETS) (2), biliary NENs are relatively rare because the mucosa lacks neuroendocrine cells and likely originates from either multipotent stem cells or neuroendocrine cells in intestinal or gastric metaplasia of the epithelium.

Given the rarity of biliary NENs, the clinicopathological characteristics and prognosis of these patients remain unclear. To date, the literature on biliary NENs is relatively sparse, and most studies are case reports (3, 4). Recently, some retrospective studies (5) with small samples have provided prognostic factors. For example, neuroendocrine carcinoma (NEC), classified by pathology, has been linked to poor prognosis in Korean patients with biliary NENs (6). Our research team also reviewed 28 biliary NENs patients in our center and found that the recurrence of the disease correlated with poor prognosis (7). Since the number of cases in each center was too small to conduct a subgroup analysis of biliary NENs, specifically focusing on the primary site of tumors (8), some studies have been performed according to national databases. For example, Cen et al. (9) selected 248 gallbladder neuroendocrine neoplasms (GB-NENs) patients from the Surveillance, Epidemiology, and End Results (SEER) database; they suggested that age, marital status, tumor size, and SEER stage are prognostic factors. However, none of these risk factors can answer the question—asked by both patients and clinicians—about survival rates, especially in regard to individual survival time. As it happens, nomogram as the graphic depictions of a statistical model that can be used to predict outcomes, and the selective advantage of the nomogram is able to provide a visual interface to aid in calculating the predicted probability that a patient will achieve a particular clinical endpoint and communication with patients.

In the present study, we sought to analyze and compare the prognostic features of biliary NENs based on a relatively large number of cases collected from the SEER database and to develop an elaborate nomogram to predict 3-year and 5-year overall survival (OS) rates based on significant prognostic factors. Further, we carried out external validation for this prediction model using our hospital database.

## Materials and Methods

### Patients

We obtained the data in this study from two sources. The first was from the SEER database. We used the SEER 18 Registries provided by the SEER*Stat Database (version 8.3.8), which consists of information on the neuroendocrine neoplasms of biliary patients (such as demographics, tumor site and morphology, tumor stage, mortality, and therapy). We derived the frequency and case distribution data from the SEER 18 Databases. The other data source was comprised of biliary NENs patients who were diagnosed with NENs and received treatment at Peking Union Medical College Hospital from 1991 to 2017. And histological assessment of tumor tissues and immunohistochemical tests were performed at the Pathology Department of Peking Union Medical College Hospital to confirm pathology and histological classification. Since SEER data are publicly available and all patient data are de-identified, institutional review board approval and informed consent were not required for this study. The included patients from our center provided oral consent and approved by the Institutional Review Board of Peking Union Medical College Hospital (S-K597). This study was performed in accordance with the 1964 Helsinki Declaration and its later amendments ethical standards.

**Figure 1:**
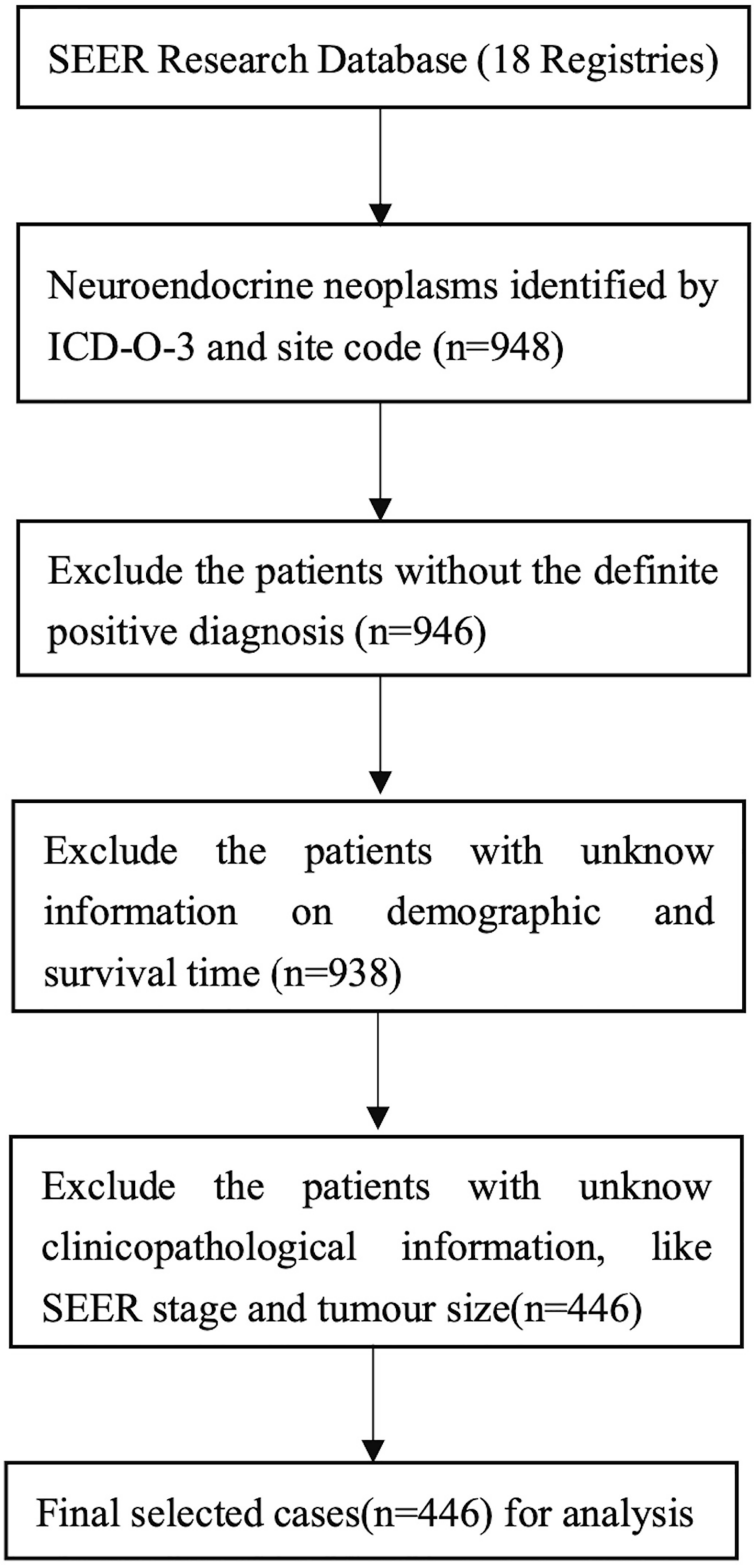
Flowchart displaying the selection procedure of NENs of biliary system cases in the SEER database.

We identified all patients with a diagnosis of neuroendocrine carcinoma, carcinoid, small cell carcinoma, large cell neuroendocrine carcinoma, and mixed adenoneuroendocrine carcinoma (MANEC) of the gallbladder, bile duct, and ampulla of Vater (AoV) using the SEER codes generated from the International Classification of Diseases for Oncology (third edition, ICD-O-3) published by the World Health Organization (WHO). The corresponding ICD-O-3 codes were 8246/3, 8240/3, 8041/3, 8013/3 and 8244/3, respectively. For the primary site of the disease, we used the topographical codes ‘C23.9, C22.1, C24.0, C24.9 and C24.1’. In addition, all included cases had a positive pathological diagnosis. We excluded patients for whom demographic or survival information was not available. **Figure 1** outlines the strategy we used to distinguish the selected cases from the SEER database.

### Data Extraction

We extracted demographic information (age, sex, race), clinicopathological characteristics (morphology/pathological classification, primary site, tumor size, SEER stage), survival time, and therapy information (surgery) from the chosen cases. We performed the pathological classification of NENs according to the 2010 ENETS/WHO criteria (10): neuroendocrine tumor (G1, G2) and neuroendocrine carcinoma (G3, small cell, large cell).

### Survival Analysis

We estimated overall survival time using the Kaplan-Meier method and long-rank test. Overall survival (OS) was defined as the period from the date of diagnosis to the date of death from various causes. Patients alive at the date of the last contact were censored. We used the univariate Cox proportional hazards model to screen out significant prognostic variables (p value<0.1) for further multivariate Cox analysis and to establish their covariate-adjusted effects on survival time. We designed all significant variables in the multivariate Cox regression (p value<0.05) and previously defined ‘variables of interest’ (site of primary tumor) as prognostic factors in the performance of nomogram construction. We carefully chose variables for inclusion to ensure parsimony of the final model.

### Nomogram Construction and Validation

For nomogram construction and external validation, we used the SEER database as the training set, and harnessed our hospital patient data set as the external validation cohort. We selected the prognostic variables for survival time *via* univariate and multivariate Cox analyses. Based on the predictive model using the identified prognostic factors, we built a nomogram to determine the 3- and 5-year OS rates. The performance of the nomogram validation included its discrimination and calibration curves through the external validation set from our hospital. We evaluated discrimination by employing a concordance index (C-index), which quantifies the probability that of two random patients, the patient who relapses first has a higher probability of the event of interest. A higher C-index indicates better discrimination. We generated a calibration plot by comparing the mean predicted survival rate with the mean actual survival rate, established through Kaplan–Meier analysis. We performed all analyses using SPSS version 25 (IBM, Armonk, NY) and R version 4.0.3. We considered p<0.05 to be statistically significant.

## Results

### Clinical Characteristics

We selected a total of 446 biliary NENs cases diagnosed between 2000 and 2017 from the SEER database. **Table 1** displays the general demographic and clinicopathological features of patients chosen from the SEER database. The majority of primary sites of biliary NENs were the gallbladder (46.4%) and AoV (41.7%). In GB-NENs, the proportion of females (65.7%) was greater than that of males, which was opposite to the NENs of the bile duct and AoV. Among the 446 NENs cases, according to the 2010 ENETS/WHO classifications, NEC (58.1%) accounted for a higher share than neuroendocrine tumors (NETs) (39.2%) and MANECs (29.7%). Regarding the SEER stage, the probability of metastasis to other organs was 22.9%. An increased number of patients exhibited a tumor size of less than 2 cm (52.9%). Most patients (80.9%) underwent operation therapy; among these operations, we classified them into three categories: partial excision, total excision, and radical surgery. More than half of the operations involved complete excision of the lesion. In addition, 28 patients from Peking Union Medical College Hospital were investigated; our colleagues have reported on their characteristics (7).

**Table 1:** Characteristics of the included patients in SEER database.

	GallbladderN=207 (%)	Bile ductN=53 (%)	Ampulla of VaterN=186 (%)	TotalN=446 (%)
**Age**				
<65 years	100 (48.3)	33 (62.3)	95 (51.1)	228 (51.1)
≥65 years	107 (51.7)	20 (37.7)	91 (48.9)	218 (48.9)
**Gender**				
Male	71 (34.3)	36 (67.9)	101 (54.3)	208 (46.6)
Female	136 (65.7)	17 (32.1)	85 (45.7)	238 (53.4)
**Race**				
White	156 (75.4)	39 (73.6)	142 (76.3)	337 (75.6)
Black	34 (16.4)	6 (11.3)	27 (14.5)	67 (15.0)
Asian/American Indian	17 (8.2)	8 (15.1)	17 (9.1)	42 (9.4)
**SEER stage**				
Localized	97 (46.9)	20 (37.7)	57 (30.6)	174 (39.0)
Regional	39 (18.8)	28 (52.8)	103 (55.4)	170 (38.1)
Distant	71 (34.3)	5 (9.4)	26 (14.0)	102 (22.9)
**Classification**				
NET	67 (32.4)	18 (34)	90 (48.4)	175 (39.2)
NEC	131 (63.3)	35 (66)	93 (30.0)	259 (58.1)
MANEC	9 (4.3)	0 (0)	3 (1.6)	12 (2.7)
**Surgery**				
No surgery	45 (21.7)	12 (22.6)	28 (15.1)	85 (19.1)
Partial excision	37 (17.9)	20 (37.7)	51 (27.4)	108 (24.2)
Total excision	106 (51.2)	8 (15.1)	36 (19.4)	150 (33.6)
Radical surgery	19 (9.2)	13 (24.5)	71 (38.2)	103 (23.1)
**Tumor size**				
≤2 cm	90 (43.5)	32 (60.4)	114 (61.3)	236 (52.9)
2˜5 cm	62 (30.3)	12 (22.6)	68 (36.2)	142 (31.8)
≥5 cm	55 (26.6)	9 (17.0)	4 (2.2)	68 (15.2)

No surgery, no surgery of primary site or autopsy only; Partial excision, simple or partial surgical removal of primary site; Total excision, total surgical removal of primary site; Radical surgery, partial or total removal of the primary site with a resection in continuity (partial or total removal) with other organs.

**Figure 2:**
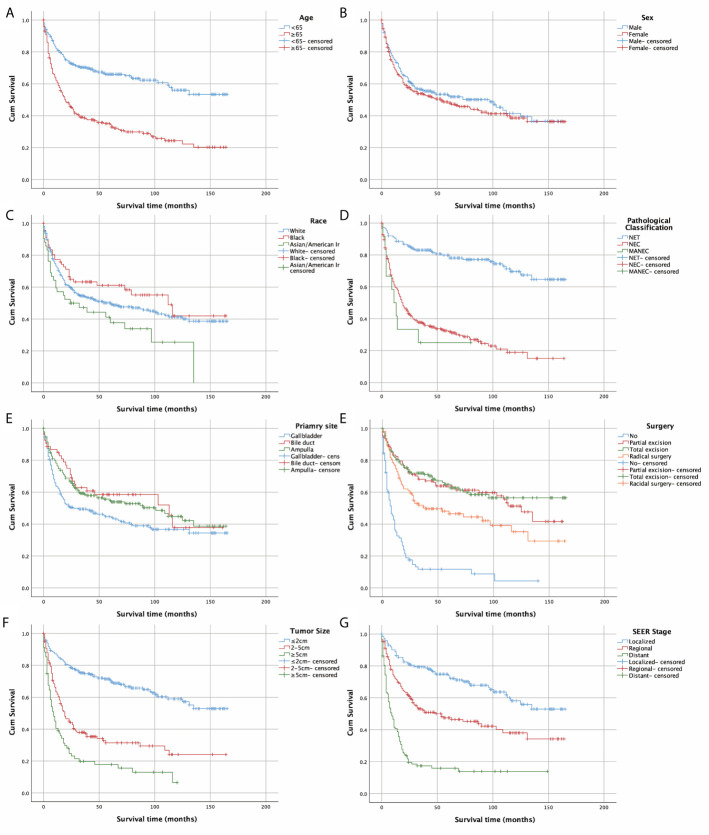
Kaplan–Meier curves of OS according to **(A)** age, **(B)** sex, **(C)** race, **(D)** pathological classification, **(E)** primary site, **(F)**surgery, **(G)** tumour size, and **(H)** SEER stage.

**Figure 2** depicts the Kaplan-Meier curves based on age, race, sex, classification, SEER stage, tumor size, primary site, and surgical options. The median OS of all included patients was 31 months. Patients younger than 65 years (43.5 months) and NETs (62 months) had longer median survival times than patients older than 65 years (19 months), neuroendocrine carcinomas (NECs) (18 months), and MANECs (12 months). With the increased severity of the SEER stage, the median survival time of patients gradually decreased (localized: regional: distant = 57: 29.5: 8.5 months). Further, patients with a smaller tumor size had better survival outcomes; the median survival time of patients with a size less than 2 cm was 55.5 months. All differences were statistically significant (p<0.001) through the long-rank test. We also found that patients with a primary site in the bile duct (median survival time: 45 months) had a significantly better outcome than patients with a primary site in the gallbladder (23 months) and AoV (33.5 months; p=0.023). Among the patients who underwent surgery, we discovered that whether the excision was partial or total had a beneficial effect on survival time. However, we did not detect increased survival time in radical surgery compared with patients who only had tumor excision.

**Table 2** presents the results of univariate and multivariate Cox analyses of biliary NENs patients from the SEER database. We regarded being older than 65, the classifications of NECs and MANECs, regional and distant stage, and the tumor size greater than 2 cm to be significant risk factors for decreased survival time. In addition, we associated the excision of primary tumors with prolonged survival time.

**Table 2:** Univariate and multivariate Cox analysis of survival time in patients selected from SEER database.

Variables	Univariate analysis		Multivariate analysis	
	HR (95%)	p	HR (95%)	p
**Sex**				
Male	1		–	
Female	1.119 (0.864-1.449)	0.309	–	–
**Age**				
<65 years	1		1	
≥65 years	2.596 (1.981-3.402)	**<0.001**	2.216 (1.677-2.930)	**<0.001**
**Race**				
White	1		–	
Black	0.794 (0.536-1.177)	0.251	0.867 (0.579-1.302)	0.495
Asian/American Indian	1.493 (1.002-2.226)	**0.049**	1.272 (0.844-1.917)	0.250
**Classification**				
NET	1		1	
NEC	4.892 (3.480-6.876)	**<0.001**	2.585 (1.748-3.822)	**<0.001**
MANEC	6.522 (3.156-13.481)	**<0.001**	2.925 (1.351-6.336)	**0.006**
**Primary site**				
Gallbladder	1		1	
Biliary tract	0.652 (0.824-1.518)	**0.056**	0.836 (0.510-1.369)	0.476
Ampulla	0.714 (0.542-0.941)	**0.017**	0.873 (0.611-1.249)	0.459
**SEER Stage**				
Localized	1		1	
Regional	2.133 (1.527-2.978)	**<0.001**	1.343 (0.917-1.968)	0.130
Distant	5.908 (4.178-8.355)	**<0.001**	2.006 (1.338-3.006)	**<0.001**
**Surgery**				
No surgery	1		1	
Partial excision	0.204 (0.140 -0.298)	**<0.001**	0.474 (0.315-0.715)	**<0.001**
Total excision	0.185 (0.129-0.265)	**<0.001**	0.398 (0.263-0.601)	**<0.001**
Radical surgery	0.320 (0.225-0.454)	**<0.001**	0.556 (0.370-0.836)	**0.005**
**Tumor size**				
≤2 cm	1		1	
2-5 cm	2.933 (2.171-3.963)	**<0.001**	1.578 (1.131-2.201)	**0.007**
≥5 cm	5.136 (3.633-7.260)	**<0.001**	1.879 (1.204-2.931)	**0.005**

The bold p value in the column of univariate analysis means that the variable was selected in the next multivariate analysis, and the bold p value in the column of multivariate analysis means that the variable was included in the construction of the nomogram.

### Nomogram Construction and Validation

**Figure 3:**
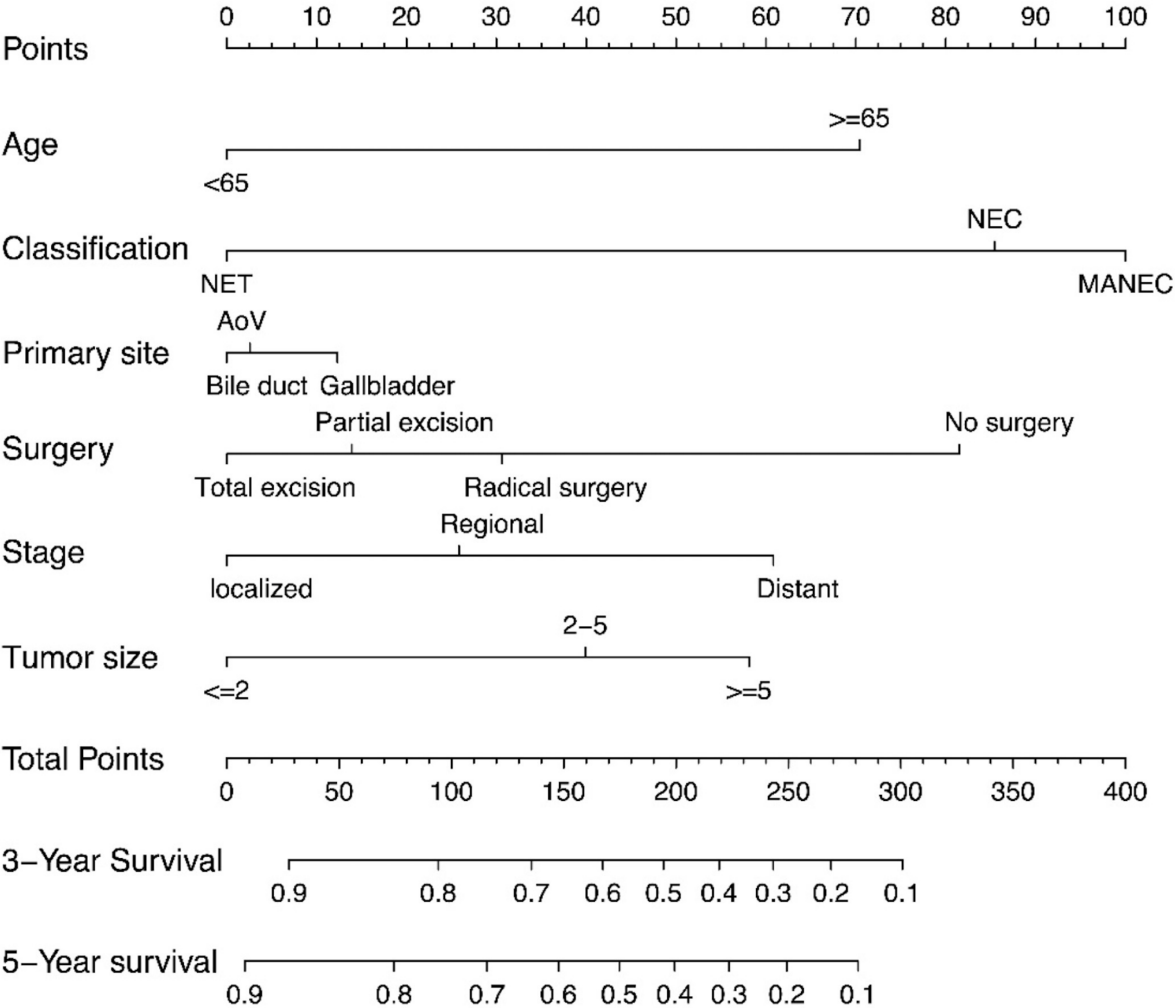
Nomograms predicting 3-year and 5-year rates of OS. Summarizing the scores of each variables together and the total points projected on the bottom scales indicate the probabilities of 3- and 5-year overall survival.

**Figure 4:**
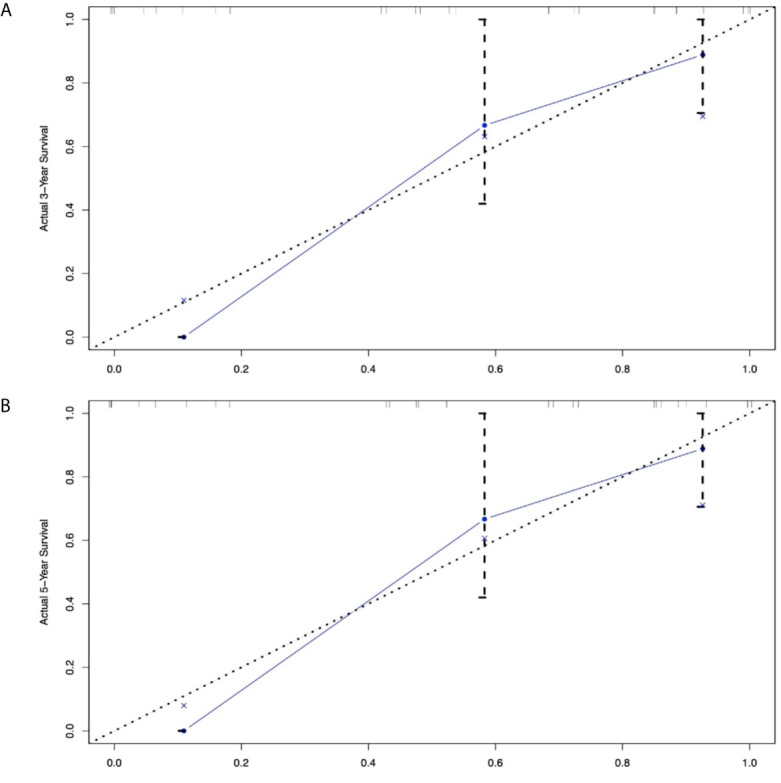
External calibration plot: **(A)** 3-year and **(B)** 5-year OS nomogram calibration curves.

In addition to the primary site (p=0.476 and 0.459)—which we previously defined as ‘variables of interest’—we recognized the following variables as prognostic factors for survival time in multivariate Cox regression analysis: age, pathological classification, stage, surgery, and tumor size. Therefore, we included all of the above variables to develop the nomogram for survival time. The nomogram can be used to predict the probability of a patient’s survival rate at 3 or 5 years (**Figure 3**). The nomogram is a graphic depiction of the model, the figure legends describe how to use the nomograms. The concordance index (C-index) of this Cox model was 0.783 (95% CI: 0.754-0.812). We performed external verification of the nomogram. The outcomes of external verification indicated that the C-index of the nomogram was 0.795 (95% CI: 0.632-0.958). **Figure 4** portrays the calibration plots for the external cross-validation at 3 years and 5 years. The x-axis represents the survival rate predicted by the nomogram, whereas the y-axis denotes the actual survival rate obtained using the Kaplan–Meier method. The findings demonstrate that the predicted 3-year and 5-year OS rates closely correspond to the actual survival rates.

## Discussion

In our study, by reviewing the clinicopathological characteristics of biliary NENs patients and exploring the prognosis and related risk factors, we developed a nomogram for the prediction of 3-year and 5-year survival rates for these patients, and performed nomogram validation using the data from our center. By using the Kaplan–Meier method and univariate and multivariate Cox analysis, we found that being older than 65 years, advanced SEER stage, increased tumor size, and pathological classification of NECs was statistically and significantly related to decreased survival time. Moreover, biliary NENs patients who underwent surgery had a better survival outcome. The developed nomogram model we used helps to easily ascertain clinical and pathological risk factors to predict the OS time for patients and physicians.

Previous studies, including case reports and literature reviews, have studied the survival time and risk factors for the prognosis of NENs with different classifications at various biliary system sites. Ayabe et al. (11) illustrated that the median OS of 300 GB-NENs, selected from NCDB Participant User Files (PUFs), was 25 months, which is similar to our result (23 months) of 207 GB-NENs. However, Karim et al. (12) reviewed the prognosis of gallbladder-NENs (GB-NENs), and discovered the median survival time to be only 9.8 months among 278 patients with GB-NENs from the SEER database, which was far below our finding. This is probably because our studies contain a certain number of well-differentiated NENs cases, which are associated with better outcomes (13, 14). Therefore, we performed subgroup analysis in these 207 cases according to pathological classification. The median survival times for NECs and MANECs were 11 months and 9 months, respectively, which is also supported by the results of Acosta et al. (15). Therefore, the pathological classification underscores this survival difference, and once again confirms that the ENETS/WHO classification is a vital prognostic factor (16). Based on this, we continued to implement subgroup analysis of NENs in the bile duct and ampulla. **Table 3** outlines the results. As the bile duct is the extremely rare primary site of NENs, with an incidence of extrahepatic bile duct NENs of 0.32% (17) among digestive system NENs, studies that focus on a statistical analysis of patient survival are usually unable to proceed, and most are case reports. However, one study (18) reported that the median survival of extrahepatic cholangiocarcinoma with neuroendocrine differentiation fluctuates between 21 and 27 months. Similar to our outcomes, the median survival of bile duct NECs was 28 months. With regard to the AoV, Randle et al. (19) indicated that the median survival of ampullary NENs was 98 months and far higher than our results (33.5 months). This difference may be due to the bias in the inclusion of patients. Hence, it is not objective to discuss the prognosis of NENs patients while ignoring the pathological classification and primary site.

**Table 3:** Median survival time (months) of different classification.

Items	Gallbladder	Bile duct	Ampulla
NET	79	78	50.5
NEC	11	28	24
MANEC	9	–	33
All	23	45	33.5

In addition to the pathological classification related to the prognosis of the biliary system, other clinical or pathological characteristics obtained in the course of diagnosis and treatment can be used to evaluate individual outcomes. For example, patients with metastasis—regardless of regional lymph nodes (20) or adjacent and distant organs—without resection of primary tumors (21) are more likely to have shorter survival times. Tumor size may also be a usable prognostic factor, but remains controversial. Kurita et al. (22) revealed that small, localized (≤ 2 cm) pancreatic neuroendocrine neoplasms have a better outcome. When the primary site was converted to the biliary system (7), tumor size was no longer a specific independent prognostic factor associated with survival time, perhaps because the diameter of the bile duct was too small to have enough space for tumor intraluminal growth. For tumor sizes of less than 1 cm, the possibility of distant metastasis is 67%, higher than that of sizes ranging between 1 and 2 cm (27.6%) and over 2 cm (28.6%) (23), so it is not recommended to predict prognosis by tumor size. However, our findings of the univariate and multivariable Cox regression analyses signal that tumor size is an independent prognostic factor. Perhaps the larger sample size and several primary sites contained could account for the differences between our results and those of prior research. Interestingly, the median survival of NENs patients who underwent radical surgery was lower than that of patients who received partial and total tumor excision. This is likely because among the 103 patients who had radical surgery, the proportion of NECs and MANECs was 66.1%, and the share of NECs and MANECs (46.9%) was lower in patients who received partial excision and total excision. One study (9) with similar results to ours showed that the combination of gallbladder surgery and lymphadenectomy had no effect on survival outcomes.

Although the multivariable Cox analysis in our research identified prognostic factors—age, SEER stage, surgery, tumor size, and pathological classification—these variables could not provide an accurate and discriminatory prediction for biliary system NENs, especially the survival rates that have been a concern for clinicians, patients, and their families. Thus, a prognostic prediction model is needed to answer these questions. For NENs, the TNM staging system (24) and ENETS/WHO classifications (25) have a certain predictive value; the former focuses on the tumor’s invasive nature, while the latter emphasizes the pathological classification. It is with great regret that confusion will likely arise from these parallel systems. The nomogram rose in response to the proper time and conditions based on these systems, including effective variables to enhance predictive ability. Regarding the nomogram in NENs, preceding studies have demonstrated its predictive value in the OS of gastric neuroendocrine neoplasms (26), the pancreas (27, 28), the small intestine (29, 30), the rectum (31), and the digestive system (32). These outcomes all signal that a specific, clinically applicable nomogram can accurately estimate the prognosis of patients with NENs. However, there is no prognostic model for biliary NENs. Our study filled this gap by creating a nomogram model to establish the OS rate of biliary NENs based on a large database. This nomogram has a predictive value with a C-index of 0.795 (95% CI: 0.632-0.958). In addition, the calibration plots of external validation, using our central database, demonstrated that the predicted 3- and 5-year OS rates closely corresponded with the actual survival rates, and verified that the nomogram exhibited excellent predictive ability. We applied the nomogram in an external validation dataset and showed that the nomogram had a good predictive value (the C-index for calibration is 0.852; 95% CI: 0.777-0.927). Hence, the nomogram can be employed to assess individual clinical outcomes more objectively.

Our study also has limitations. A major constraint is that our nomogram was created using just six clinicopathological factors, lacking other additional variables such as Ki-67 (33, 34), and the high Ki-67 index is associated with portal venous tumor invasion which is a prognostic factor for patients with pancreatic NENs (35). Besides, lactate dehydrogenase (LDH) has been found to increase in patients with NEC (36). However, these variables are not available in the SEER database and it’s necessary to incorporate LDH level and Ki-67 into analysis in further investigation. Another obstacle is that the sample size of the validation cohort was small, and only one center was included. Although the verification results and power analysis (Power = 0.8689) were good, the value of the C-index may change after adding samples or centers. Future studies could include validation cohorts from different centers to control for selection bias to some extent. Besides, in terms of treatment of NENs, we only considered the effects of surgery on prognosis, ignoring neoadjuvant or adjuvant therapy (37), as well as other medical therapies, like somatostatin analogues, peptide receptor radionuclide therapy (38) and target therapies. The last limitation is that we regard the AoV as part of the biliary system, but from an anatomical angle, the AoV is the junction between the bile duct and the pancreatic duct. The SEER database did not provide anatomical information on AoV. With the accumulation of cases, subgroup analysis could be performed in future studies according to the primary location of the disease.

## Conclusion

In conclusion, age, tumor size, pathological classification, surgery, and SEER stage are predictors for the survival of biliary NENs patients. We established and externally validated an unprecedented nomogram to determine the prognoses of patients with biliary NENs. Because our nomogram included only six common clinicopathological variables, it can be used as a potentially objective clinical tool for physicians to predict the prognosis of these patients around the world.

## Data Availability Statement

The raw data supporting the conclusions of this article will be made available by the authors, without undue reservation.

## Ethics Statement

The studies involving human participants were reviewed and approved by Institutional Review Board of Peking Union Medical College Hospital. Written informed consent for participation was not required for this study in accordance with the national legislation and the institutional requirements.

## Author Contributions

SZ participated in the design of this study and wrote the manuscript. XH conceived the original idea, supervised the overall direction and planning of the project. SZ, SJ, HY, LD, WC, SH, and HZ contributed to the acquisition of the data, analysis, and interpretation of the data. All authors contributed to the article and approved the submitted version.

## Funding

This study was funded by CAMS Innovation Fund for Medical Sciences (CIFMS, 2017-I2M-4-003), and Program Focus Health of Liver and Gallbladder in Elder (ZYJ201912).

## Conflict of Interest

The authors declare that the research was conducted in the absence of any commercial or financial relationships that could be construed as a potential conflict of interest.
