# Supplementary material for: Biliary Neuroendocrine Neoplasms: Analysis of Prognostic Factors and Development and Validation of a Nomogram
Source: Front Oncol. 2021 Jul 19;11:654439. doi: 10.3389/fonc.2021.654439 (PMC8327779; doi:10.3389/fonc.2021.654439)
Supplement: Supplementary file 1 [file DataSheet_1.pdf]

S Table 1. Power analysis of COX Regression in Validation cohort

Variables	Power	Sample Size (N)	Reg. Coef. (B)	S.D. of X1 (SD)	Event Rate (P)	R-squared X1 vs Other X's (R^2)	One-side Alpha	Beta
**Size**	**0.8689**	**28**	**0.6307**	**1.1790**	**0.5350**	**0.0760**	**0.0500**	**0.1311**
**Surgery**	**0.7191**	**28**	**-0.9213**	**0.6120**	**0.5350**	**0.2720**	**0.10000**	**0.2809**
**SEER Stage**	**0.5477**	**28**	**0.6960**	**0.5670**	**0.5350**	**0.1580**	**0.10000**	**0.4523**
**Primary Site**	**0.2387**	**28**	**-0.1791**	**0.9370**	**0.5350**	**0.2270**	**0.10000**	**0.7613**
**Classification**	**0.4162**	**28**	**0.9497**	**0.3150**	**0.5350**	**0.1460**	**0.10000**	**0.5838**
**Age**	**0.4409**	**28**	**0.7957**	**0.3900**	**0.5350**	**0.1100**	**0.10000**	**0.5591**

B: This value is the log (base e) of the hazard ratio; Alpha: The probability of obtaining a false positive with the statistical test.

S Table 2. Sample size of COX Regression in Validation cohort

Variables	Power	Sample Size (N)	Reg. Coef. (B)	S.D. of X1 (SD)	Event Rate (P)	R-squared X1 vs Other X's (R^2)	One-side Alpha	Beta
Surgery	0.8048	37	-0.9213	0.6120	0.5350	0.2720	0.10000	0.1952
SEER Stage	0.8034	65	0.6960	0.5670	0.5350	0.1580	0.10000	0.1966
Primary Site	0.8007	388	-0.1791	0.9370	0.5350	0.2270	0.10000	0.1993
Classification	0.8020	111	0.9497	0.3150	0.5350	0.1460	0.10000	0.1980
Age	0.8020	99	0.7957	0.3900	0.5350	0.1100	0.10000	0.1980
