# Supplementary material for: Biliary Neuroendocrine Neoplasms: Analysis of Prognostic Factors and Development and Validation of a Nomogram
Source: Front Oncol. 2021 Jul 19;11:654439. doi: 10.3389/fonc.2021.654439 (PMC8327779; doi:10.3389/fonc.2021.654439)
Supplement: Supplementary file 2 [file DataSheet_2.pdf]

Fig.1

☐ **Aged**

1. A person 65 through 79 years of age. For a person older than 79 years, **AGED**, 80 AND OVER is available.
Year introduced: 1966

☐ Middle **Aged**

3. An adult **aged** 45 - 64 years.
Year introduced: 2004(1966)

Fig.2

2010 ENETS/WHO nomenclature and classification for neuroendocrine neoplasms arising in the gastrointestinal (GI) tract

Differentiation	Grade	Mitotic count*	Ki-67 index¶	Traditional	ENETS, WHO
Well differentiated	Low grade (G1)	<2 per 10 HPF	<3%	Carcinoid, islet cell, pancreatic (neuro)endocrine tumor	Neuroendocrine tumor, G1
	Intermediate grade (G2)	2 to 20 per 10 HPF	3 to 20%	Carcinoid, atypical carcinoidΔ, islet cell, pancreatic (neuro)endocrine tumor	Neuroendocrine tumor, G2
Poorly differentiated	High grade (G3)	>20 per 10 HPF	>20%	Small cell carcinoma	Neuroendocrine carcinoma, G3, small cell
				Large cell neuroendocrine carcinoma	Neuroendocrine carcinoma, G3, large cell

ENETS: European Neuroendocrine Tumor Society; WHO: World Health Organization; HPF: high-power fields.
* Counted in 10 HPF; 10 HPF = 2 mm^2, at least 40 fields (at 400x magnification) evaluated in areas of highest mitotic density. Cut-offs per American Joint Commission on Cancer Staging Manual, 8th edition.
¶ Ki-67 index as assessed by MIB1 antibody staining: percent positive after count of 2000 cells in area of highest nuclear labeling. Cut-offs per American Joint Commission on Cancer Staging Manual, 8th edition.
Δ The term "atypical carcinoid" only applies to intermediate-grade neuroendocrine tumors of the lung.

Graphic 60365 Version 15.0
